# Inter-element miscibility driven stabilization of ordered pseudo-binary alloy

**DOI:** 10.1038/s41467-022-28710-0

**Published:** 2022-02-24

**Authors:** Kenshi Matsumoto, Ryota Sato, Yasutomi Tatetsu, Ryo Takahata, Seiji Yamazoe, Miho Yamauchi, Yuji Inagaki, Yoichi Horibe, Masaki Kudo, Takaaki Toriyama, Mitsunari Auchi, Mitsutaka Haruta, Hiroki Kurata, Toshiharu Teranishi

**Affiliations:** 1 Institute for Chemical Research, Kyoto University, Gokasho, Uji, Kyoto, 611-0011 Japan; 2 Center for Liberal Arts Education, Meio University, Biimata, Nago, Okinawa, 905-8585 Japan; 3 Department of Chemistry, Graduate School of Science, Tokyo Metropolitan University, 1-1 Minami-Osawa, Hachioji, Tokyo, 192-0397 Japan; 4 Institute for Materials Chemistry and Engineering (IMCE), Kyushu University, 744 Motooka, Nishi-ku, Fukuoka, 819-0395 Japan; 5 Department of Applied Quantum Physics and Nuclear Engineering, Faculty of Engineering, Kyushu University, 744 Motooka, Nishi-ku, Fukuoka, 819-0395 Japan; 6 Department of Materials Science and Engineering, Graduate School of Engineering, Kyushu Institute of Technology, 1-1 Sensui, Tobata, Kitakyuushu, Fukuoka, 804-8550 Japan; 7 The Ultramicroscopy Research Center, Kyushu University, 744 Motooka, Nishi-ku, Fukuoka, 819-0395 Japan

**Keywords:** Nanoparticles, Synthesis and processing, Nanoparticles, Synthesis and processing

## Abstract

An infinite number of crystal structures in a multicomponent alloy with a specific atomic ratio can be devised, although only thermodynamically-stable phases can be formed. Here, we experimentally show the first example of a layer-structured pseudo-binary alloy, theoretically called *Z*3-FePd_3_. This *Z*3 structure is achieved by adding a small amount of In, which is immiscible with Fe but miscible with Pd and consists of an alternate *L*1_0_ (CuAu-type)-PdFePd trilayer and Pd–In ordered alloy monolayer along the **c** axis. First-principles calculations strongly support that the specific inter-element miscibility of In atoms stabilizes the thermodynamically-unstable *Z*3-FePd_3_ phase without significantly changing the original density of states of the *Z*3-FePd_3_ phase. Our results demonstrate that the specific inter-element miscibility can switch stable structures and manipulate the material nature with a slight composition change.

## Abstract (web summary)

Synthetic routes of stabilizing crystal structures can discover atomic pickings with desired properties. Here the authors demonstrate inter-element miscibility of In can act as a stabilizer to create Z3-based ordered alloy without significantly changing the original density of state of Z3-FePd3.

## Introduction

The crystal structure is a crucial factor for determining the physical and chemical properties because the atomic arrangement dominates the atomic-orbital hybridization^1–5^. In a multicomponent alloy system, even with a specific atomic ratio, a number of geometrically available crystal structures can be devised^6,7^. Although this geometrical possibility means that there is still room to investigate crystal-structure control to realize a fine or drastic tuning of material properties, only thermodynamically stable phases at low and high temperatures have been commonly formed which follow the phase diagrams^8^, except for the recently reported cases under extreme conditions, such as size^9^, pressure^10^, and lattice strain^11^. For instance, in the case of the FePd_3_ alloy system targeted in this research, only the thermodynamically stable *L*1_2_ (Cu_3_Au-type) phase has been experimentally obtained^5,7,8^. Therefore, a synthetic strategy of stabilizing a crystal structure is deserved.

To create an ordered alloy with such an atomic arrangement as the layered structures shown in Supplementary Fig. 1, we paid attention to the inter-element miscibility—the miscibility between two elements at an atomic level—in a binary-alloy phase diagram^8^. Recently, the transformation from *A*1- to *L*1_0_-FeNi structures was achieved by introducing and withdrawing interstitial N atoms^12^. The Fe–Ni–N compound formed during this process possessed a crystal structure with a unit cell composed of one Fe–N layer sandwiched by two Ni layers in such a way as to reflect the better binding affinity of Fe with N, such as N_2_ dissociation by Fe catalyst^13^. This structural transformation caused by the interelement affinity of an interstitial N with Fe and Ni inspired us to analogically consider that the transformation from well-known to unexplored structures of a binary alloy could be achieved by the difference in the miscibility of a substitutional third element with the two kinds of elements in binary alloys.

To demonstrate this idea, we focused on the binary alloy of Pd and Fe, because many third elements can be miscible with Pd and immiscible with Fe in the binary phase diagram^8^. The element In was selected as a substitutional third element. Between the well-known *L*1_2_-FePd_3_ and *L*1_0_-FePd phases in the Fe–Pd system, we chose the *L*1_2_-FePd_3_ as a target crystal phase because of its likeliness to change crystal structure from an isotropic *L*1_2_ structure to a highly anisotropic structure when selectively locating the In atoms at specific sites. Such a structural change would, in turn, alter the physical properties. However, according to previous reports, Fe–Pd–In ternary alloys have been rarely reported except for the In-rich ternary alloys (In/(Fe+Pd+In) >20 atomic percent (at.%))^14,15^ and the In-poor *L*1_2_-type FePd_3_ ternary alloy phases (In/(Fe+Pd+In) <1.2 at.%)^16^. This indicates the difficulty in producing other Fe–Pd–In ternary alloys with a small amount of In composition (e.g., 1.2 < In/(Fe+Pd+In) < 20 at.%).

In this work, we create the Fe–Pd–In ternary alloy phase (11 < In/(Fe+Pd+In)< 14 at.%) with a *Z*3-based structure by using nanoparticulate precursor powders, because the uniformly mixing of Fe, Pd, and In at the nanoscale is crucial for the formation of *Z*3-type structure. Experimental results and first-principles calculation show that the stabilization of *Z*3-Fe(Pd,In^*d*^)_3_ is induced by not a nanosize or a kinetic effect but a specific inter-element miscibility of In, which is miscible with Pd but immiscible with Fe. This idea that the inter-element miscibility of the third element works as a stabilizer of an ordered structure gives us an opportunity for the experimental discovery of a variety of novel ordered structures.

## Results

### Creation of layer-structured Fe–Pd–In alloy nanoparticles

**Fig. 1:**
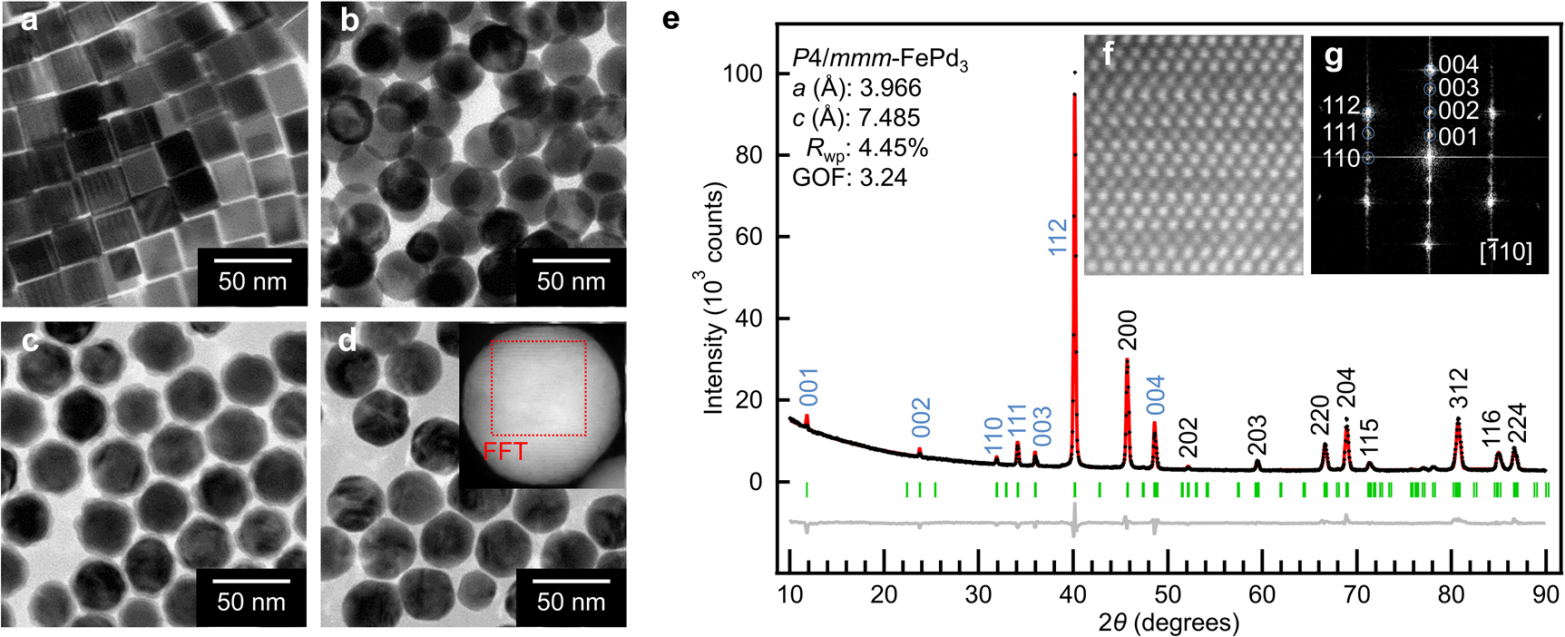
Synthesis and characterization of monodisperse Fe–Pd alloy NPs with a small amount of In. **a**–**d**, TEM images of 23-nm Pd NPs (**a**), *A*1-PdIn_*x*_ NPs with Pd/In = 82/18 at.% (**b**), *A*1-PdIn_*x*_@FeO_*y*_ core@shell NPs with Pd/Fe = 73/27 at.% (**c**) and the NPs obtained by the reductive annealing of **c** at 600 °C for 3 h (**d**) (the inset shows the HAADF-STEM image of **d**). **e**, Rietveld refinement for powder XRD patterns of the NPs obtained by the reductive annealing of **c** at 800 °C for 3 h, where the black markers are raw data, the red is the fitting curve, the gray line is a difference curve of raw data and the fitting curve, and the green bars stand for the whole diffraction-peak positions. *R*_wp_ and GOF are a reliability factor and a goodness of fit, respectively. **f**,**g**. Magnified HAADF-STEM image (**f**) and the fast Fourier transform (FFT) image (**g**) of the inset in **d**.

**Fig. 2:**
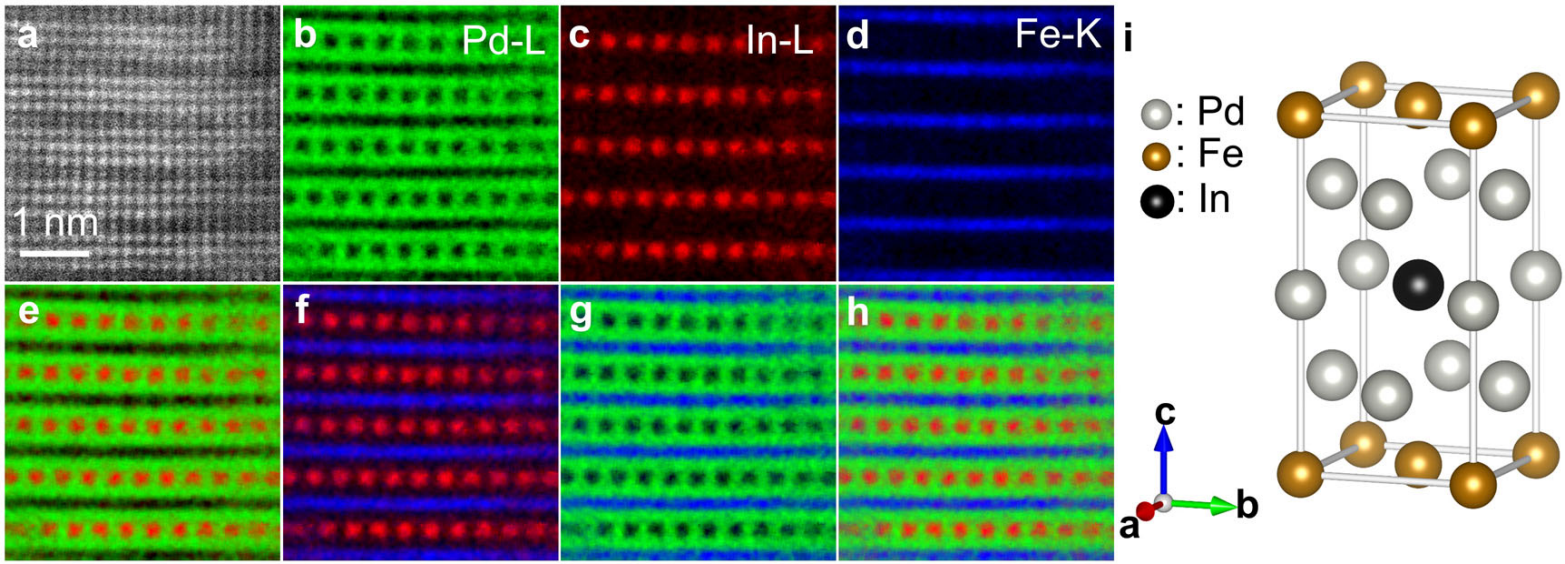
Direct observation of In atoms in *Z*3-type structure. **a**–**d**, HAADF-STEM image (**a**) and the elemental maps (Pd-L (**b**), In-L (**c**) and Fe-K (**d**)) of *Z*3-type structure. **e**–**h**, Overlay of **b** and **c** (**e**), **c** and **d** (**f**), **b** and **d** (**g**), and **b**–**d** (**h**). **i**, Unit cell of *Z*3-Fe(Pd,In^*d*^)_3_ determined from Fig. 1e–g and Fig. 2a–h.

Fe–Pd alloy nanoparticles (NPs) containing a small amount of In atoms were synthesized by a step-by-step chemical synthesis (Supplementary Table 1); (i) synthesis of 23-nm Pd NPs (Fig. 1a)^17^, (ii) alloying of In with Pd NPs to form *A*1-PdIn_*x*_ NPs (Fig. 1b), (iii) growth of FeO_*y*_ shells on *A*1-PdIn_*x*_ NPs (Fig. 1c)^17,18^ and (iv) diffusion of Fe atoms into *A*1-PdIn_*x*_ NPs through the reductive annealing of FeO_*y*_ shells at 600 °C or 800 °C for 3 h under a 4% H_2_ gas flow (Ar balance) (Fig. 1d). First, we analysed the NPs with the Pd/In/Fe atomic ratio of 63/14/23 (Pd/In = 82/18 at.% and Pd/Fe = 73/27 at.%), which was confirmed by energy-dispersive X-ray spectrometry (EDX). Figure 1e–g show Rietveld refinement for the powder X-ray diffraction (XRD) patterns and the high-angle annular dark-field scanning transmission electron microscopy (HAADF-STEM) image of the final NPs. These NPs could not be assigned to any structures in the Fe−Pd and Pd−In systems^8^, but it was found that they had the *P*4/*mmm* structure with the atomic coordinates of Fe^1*a*^ (0, 0, 0), Fe^1*c*^ (0.5, 0.5, 0), Pd^4*i*^ (0.5, 0, 0.23), Pd^1*b*^ (0, 0, 0.5), and Pd^1*d*^ (0.5, 0.5, 0.5) in the unit cell, where the superscripts refer to the multiplicities and Wyckoff letters. Therefore, the NPs had a superlattice structure with an alternate stacking of a single Fe-atomic layer and three Pd-atomic layers along the **c** axis (Fig. 1e–g), which is called the *Z*3-FePd_3_^6,7,19^. However, it was quite difficult to detect the In atoms from the intensity ratio of the XRD peaks and the *Z* contrast of the HAADF-STEM image, because the electron density of In is very close to that of Pd. To identify the In sites in the *Z*3 framework, we performed elemental mapping by EDX at the atomic resolution. As a result, In atoms were found to be selectively excluded from both the Fe and In atoms and preferentially occupied the 1*d* or 1*b* site, which corresponded to the middle Pd layer among the three Pd layers (Fig. 2a–e). Consequently, the resulting *Z*3 structure with the In atoms at the 1*d* site was precisely denoted to be *Z*3-Fe(Pd,In^*d*^)_3_.

### Dependence of Fe–Pd–In alloy phase on In quantity

Next, to investigate the In composition inducing the formation of the *Z*3-type structure, the Pd–In@FeO_*y*_ core@shell NPs (Pd/Fe ≈ 70/30 at.%) were annealed at 800 °C for 3 h (Supplementary Table 1). Powder XRD patterns verified that the reductive annealing for Pd–In@FeO_*y*_ core@shell NPs with the Pd/In of 89/11 at.%, 85/15 and 83/17 at.%, and 73/27 at.% gave the *L*1_2_-type phase, the *Z*3-type phases and a mixture of all three phases (*A*1-Fe_60_Pd_40_, *Pnma*-PdIn_2_, and *I*4/*mmm* phases), respectively (Supplementary Fig. 2). This indicated that the *Z*3-type structure can be formed in a narrow range of In compositions.

To further confirm the In sites in not only the *Z*3-type but also the *L*1_2_-type structures, we performed the X-ray absorption fine structure (XAFS) measurement for these structures at the Fe-, Pd- and In-K edges. The In environment determined from the extended XAFS (EXAFS) regions at the In-K edge revealed that the In atoms in the *L*1_2_-type and *Z*3-type structures were surrounded only by approximately 12 Pd atoms, that is, *L*1_2_-(Fe,In)Pd_3_ and *Z*3-Fe(Pd,In^*d*^)_3_ were formed by partially replacing Fe and Pd (1*d* site) far from the Fe with In, respectively (Supplementary Fig. 3 and Table 2). This tendency strongly implied that the inter-element miscibility of In with other elements, that is, immiscible with Fe and miscible with Pd, dominated the In sites.

### Investigation of driving force for stabilizing *Z*3-type structure

Although we found that the formation of *Z*3-type structure depends on the In composition, the driving force for stabilizing *Z*3-type structure is yet to be clarified. Besides the thermodynamic effect, the kinetic and/or the nano-size effects^9^ should be considered as the candidates of the driving force for forming *Z*3-type structure. First, to investigate whether *Z*3-type structure was a kinetically or a thermodynamically stable phase below 800 °C, we conducted in situ XRD measurement for *A*1-PdIn_*x*_@FeO_*y*_@SiO_2_ nanoparticles under the reductive annealing at 800 °C (SiO_2_ was coated on *A*1-PdIn_*x*_@FeO_*y*_ nanoparticles to avoid the interparticle fusion). As a result, only *Z*3-type phase was formed by the reductive annealing at 800 °C for 4 min and further annealing at 800 °C for 25 h did not change the crystal phase (Supplementary Fig. 4). Therefore, we concluded that the *Z*3-type structure was a thermodynamically stable phase below 800 °C.

Next, to investigate whether a particle size contributes to stabilizing the *Z*3-type structure, we synthesized micrometer-scale Fe–Pd–In ternary alloy particles via the interparticle sintering of PdIn@FeO_*y*_ nanoparticles by the reductive annealing at 800 °C for 3 h, which gave the *Z*3-type structure (Supplementary Fig. 5). On the other hand, when the reductive annealing was conducted for a mixture of Pd@FeO_*y*_ NPs (Pd/Fe = 74/26 at.%) and In powder (Pd/In = 82/18 at.%), in which In was inhomogeneously distributed, at 800 °C for 3 h, the *Z*3-type structure did not form (Supplementary Fig. 6). These results strongly support that the nanoparticulate precursor powders composed of homogeneously mixed Fe, Pd, and In at the nanoscale are crucial for the formation of *Z*3-type structure even if the *Z*3-type structure is not stable specifically at the nanoscale^9^.

**Fig. 3:**
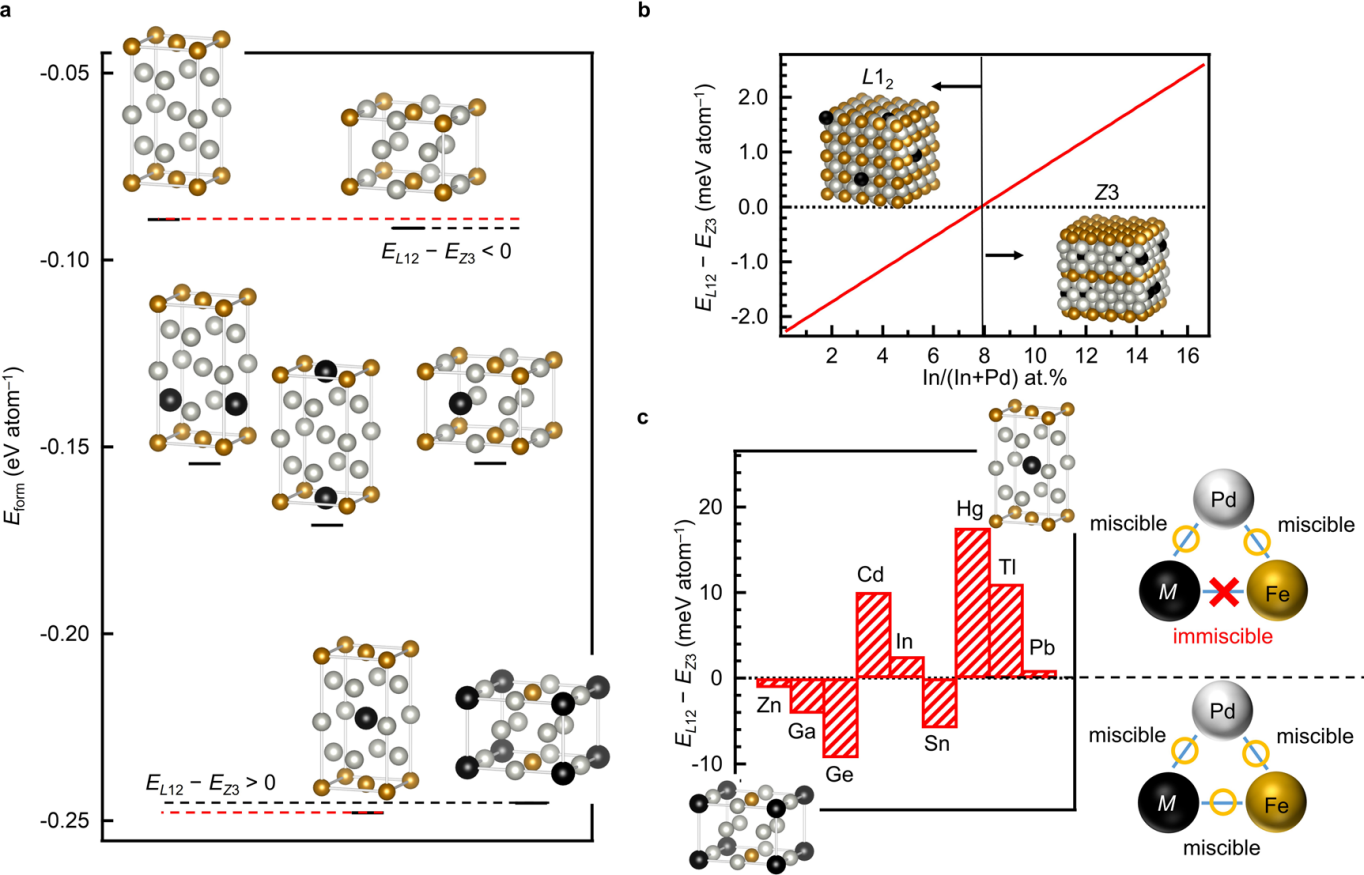
Formation energies of *Z*3-type versus *L*1_2_-type structures. **a** Formation energies of *Z*3-type and *L*1_2_-type phases (*E*_form_) with In atoms in different sites. **b**, In composition-dependent formation energy difference between *Z*3-Fe_2_(Pd_6-*x*_,In_*x*_^*d*^) and *L*1_2_-(Fe_2-*x*_,In_*x*_)Pd_6_ (0 ≤ *x* ≤ 1) (*E*_*Z*3_ and *E*_*L*12_, respectively) phases, where 16.67 of In/(In+Pd) at.% corresponds to *x* = 1. **c**, Formation energies of most stable *Z*3-Fe_2_(Pd_5_,*M*_1_^*d*^) versus most stable *L*1_2_-(Fe_1_,*M*_1_)Pd_6_ in each crystal structure with the 12–14 groups additional-metallic element (*M*). Only when adding the elements *M* immiscible with Fe, was the *Z*3-type structure more stable than the *L*1_2_-type structure.

To determine why the *Z*3-Fe(Pd,In^*d*^)_3_ structure was preferentially formed, we undertook a theoretical approach using first-principles calculations. First, we calculated the formation energies (*E*_form_) of various FePd_3_ phases in *L*1_2_ and *Z*3 structures before and after adding In by using the equation below;

1 $${E}_{{{{{{\rm{form}}}}}}}=E[{{{{{{\rm{Fe}}}}}}}_{x}{{{{{{\rm{Pd}}}}}}}_{y}{{{{{{\rm{In}}}}}}}_{z}]-(x\times \mu [{{{{{\rm{Fe}}}}}}]+y\times \mu [{{{{{\rm{Pd}}}}}}]+z\times \mu [{{{{{\rm{In}}}}}}])$$

where *E*[Fe_*x*_Pd_*y*_In_*z*_] represents the total energies of *Z*3- or *L*1_2_-Fe_*x*_Pd_*y*_In_*z*_ ((*x*, *y*, *z*) = (2, 6, 0), (2, 5, 1), or (1, 6, 1)) and μ[Fe], μ[Pd] and μ[In] are the chemical potentials of Fe, Pd and In, respectively, which are equivalent to their total energies of the ground states. As shown in Fig. 3a, the calculation results showed that the *L*1_2_-(Fe_1_,In_1_)Pd_6_ and *Z*3-Fe_2_(Pd_5_,In_1_^*d*^) structures were most stable in each structural type, which was in good agreement with the In sites in *L*1_2_-type and *Z*3-type structures estimated from XAFS analysis. Furthermore, the differences in *E*_form_ values between *L*1_2_-(Fe_2-*x*_,In_*x*_)Pd_6_ and *Z*3-Fe_2_(Pd_6-*x*_,In_*x*_^*d*^) (0 < *x* < 1) (see Methods) showed that the *Z*3-type structure became more stable than the *L*1_2_-type structure from *x* > 0.48, or In/(In+Pd) > 8 at.% (Fig. 3b). The calculation results also agreed with the experimental tendency in terms of the phase transition and the critical In/Pd at.% from the *L*1_2_-type to *Z*3-type phases when increasing the In composition. Therefore, although the *L*1_2_-FePd_3_ structure is thermodynamically stable for FePd_3_ systems^5,7,8^, introducing a small amount of In atoms into FePd_3_ systems makes the *Z*3-type structure more stable than the *L*1_2_-type structure.

The In sites shown in the experimental and theoretical data were likely to reflect the inter-element miscibility of In with Fe and Pd. To investigate whether the inter-element miscibility of In contributed to the stabilization of *Z*3-type phase, we conducted the same first-principles calculations for the nine 12–14 groups elements (*M* = Zn, Cd, Hg, Ga, In, Tl, Ge, Sn, and Pb) in the periodic table as that used for In. Very interestingly, the *Z*3-type structure was more stable than the *L*1_2_-type structure when introducing Cd, Hg, In, Tl and Pb which possessed the same miscibility as In with Fe and Pd^8,20^. On the other hand, the *Z*3-type structure remained unstable when adding Zn, Ga, Ge, and Sn which are miscible with Fe and Pd^8^ (Fig. 3C). Since these calculations do not consider other candidates except for isotropic *L*1_2_-type and anisotropic *Z*3-type phases, one should note the differences in these formation energies at this composition, however, in the Fe–Pd–Pb system, the formation of *Z*3-Fe(Pd_2.5_,Pb_0.5_^*d*^) structure was confirmed by the similar step-by-step chemical synthesis as the synthetic process inducing *Z*3-Fe(Pd,In^*d*^)_3_ structure (see Methods and Supplementary Fig. 7). These results strongly support our claim that a third element selected by specific inter-element miscibility possess the potential to stabilize a binary alloy like the *Z*3 structure.

### Physical properties of *Z*3-Fe(Pd,In^*d*^)_3_ structure

**Fig. 4:**
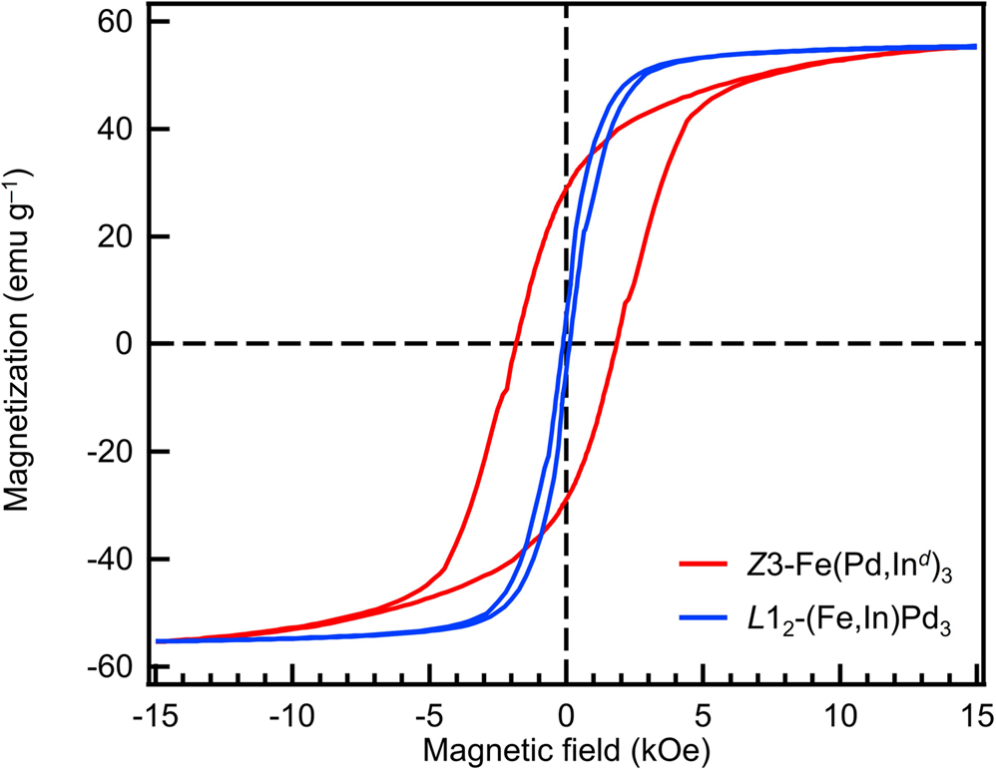
Magnetic properties of *L*1_2_-(Fe,In)Pd_3_ and *Z*3-Fe(Pd,In^*d*^)_3_ NPs. *M*–*H* hysteresis loop of *Z*3-type NPs (Pd/In = 83/17 at.%) and *L*1_2_-type NPs (Pd/In = 89/11 at.%) measured by VSM at room temperature.

Finally, we investigated the novel physical properties characteristic of the *Z*3-type structure. First, the *Z*3-type structure was expected to show high coercivity because of having a *L*1_0_-type PdFePd trilayer^17,21,22^. Magnetization–magnetic field (*M*–*H*) curves were measured for the *L*1_2_-(Fe,In)Pd_3_ and *Z*3-Fe(Pd,In^*d*^)_3_ NPs (Pd/Fe ≈ 70/30 at.%) synthesized by annealing at 600 °C for 3 h with the Pd/In at.% of 89/11 and 83/17, respectively, at room temperature by means of a vibrating sample magnetometer (VSM). Both the *L*1_2_-type and *Z*3-type NPs showed a ferromagnetic feature with almost the same saturation magnetization, while the *Z*3-type NPs possessed a coercivity that was 15 times higher than the *L*1_2_-type NPs and behaved as a magnetically hard phase similar to the *L*1_0_ NPs^21,22^ (Fig. 4). This coercivity enhancement could be explained by the drastic increase in the magnetic anisotropy energy^23^ induced by the crystal structure change from *L*1_2_ to *Z*3 frameworks (−1.38 μeV atom^–1^ for *L*1_2_-type structure and −0.213 meV atom^–1^ for *Z*3-type structure (see Methods)).

As another structure-specific physical property, the *Z*3-type structure is also likely to possess a hydrogen-storage ability, owing to one Pd–In ordered alloy layer sandwiched by two Pd layers similar to the Pd-rich alloy structure^24^. However, it was found from the pressure-composition isotherms at 30 °C and 60 °C that this *Z*3-type structure had no hydrogen-storage ability (Supplementary Fig. 8). This hydrogen-storage ability can be understood by the small amount of 4*d* hole of Pd in the *Z*3-type structure shown by the density of states (DOS), which are obtained from first-principles calculations (Supplementary Fig. 9)^25^. The DOS feature also showed no significant difference in the DOS near the Fermi level and the *d*-bandwidths and centers of Fe and Pd between the *Z*3-Fe_2_(Pd_5_,In_1_^*d*^) and *Z*3-Fe_2_Pd_6_ structures. No significant change in DOS of *Z*3-Fe_2_(Pd_5_,In_1_^*d*^) and *Z*3-Fe_2_Pd_6_ structures indicates that the physical properties characteristic of the *Z*3-FePd_3_ structure are still observed even if a small amount of In atoms are introduced into the *Z*3-FePd_3_ framework.

## Discussion

We proposed the concept that the inter-element miscibility stabilizes a binary alloy with an ordered structure. As an example, we demonstrated that introducing a small amount of In atoms enabled the formation of *Z*3-Fe(Pd,In^*d*^)_3_ NPs with an almost identical DOS as the *Z*3-FePd_3_ structure, where the synthetic procedure using the nanoparticulate precursor powders such as *A*1-PdIn_*x*_@FeO_*y*_ NPs were crucial for creating the *Z*3-type structure. These results indicate that ordered alloy structures can be experimentally discovered according to the binary-phase diagrams^8,20^ rather than searching for synthesizable structures using first-principles calculations^26,27^. We expect that a variety of ordered alloy nanostructures will be discovered that exhibit unexpected and excellent properties.

## Methods

### Materials

All reagents and solvents were commercially available. Sodium tetrachloropalladate(II) (Na_2_PdCl_4_, 98%), polyvinylpyrrolidone (PVP, *M*_w_ ≈ 55,000), ascorbic acid (AA, 99 + %), potassium bromide (KBr, ≥ 99%), oleylamine (OAm, 80–90%), 1-octadecene (ODE, 90%), indium(III) chloride (InCl_3_, 98%), indium powder (In, >99.99%), eicosane (99%), ammonium hydrogen solution (NH_3_aq, 28–30%), tetraethyl orthosilicate (TEOS, 98%) and polyoxyethylene nonylphenylether (IGEPAL@CO-520, *M*_w_ = 441) were purchased from Sigma-Aldrich. Iron pentacarbonyl (Fe(CO)_5_, 95%) was purchased from Kanto Chemical Corporation. Oleic acid (OA, > 85%) was purchased from Tokyo Chemical Industry. Lead (II) acetate trihydrate (Pb(OAc)_2_·3H_2_O), ethanol, acetone, *n*-hexane, chloroform, and cyclohexane were purchased from Wako Pure Chemical Industries. Boron nitride (BN, > 99.5%) powder was purchased from Nacalai Tesque. Chemical reagents were used as received without further purification except for the synthetic process of Pd–Pb@FeO_*y*_ NPs.

### Synthesis of Pd NPs

Pd NPs were synthesized by the previously reported procedure^17^. After the rapid injection of an aqueous solution (30 mL) of Na_2_PdCl_4_ (1.5 g) and KBr (8.0 g) into an aqueous solution (270 mL) of PVP (2.8 g) and AA (0.66 g), the reaction solution was kept at 80 °C for 3 h and then cooled to room temperature. The above black solution of Pd seeds (15 mL) was injected into an aqueous solution (250 mL) of Na_2_PdCl_4_ (1.5 g) and KBr (3.0 g) at 40 °C and then an aqueous solution (25 mL) of PVP (2.8 g) and an aqueous solution (25 mL) of AA (1.8 g) were injected every 30 min at 40 °C. This solution was kept at 40 °C for 48 h, heated from 40 °C to 60 °C and kept at 60 °C for 48 h. Finally, the reaction solution was cooled to room temperature and the Pd NPs with an average edge length of 23 nm were collected by centrifugation with acetone and then purified with an ethanol/acetone (1/4 vol.%) mixed solvent.

### Synthesis of Pd–In alloy NPs

To react the Pd NPs with the In precursor in an organic solution, the surface ligand passivating the Pd NPs was exchanged from PVP to OAm by heating a mixture of OAm (10 mL), chloroform (20 mL) and Pd NPs (0.5 mmol) at 50 °C for 30 min. The solution was cooled to room temperature and the OAm-protected Pd NPs were collected by centrifugation with ethanol and purified twice with a chloroform/ethanol (2/3 vol.%) mixed solvent. After the ligand exchange, the OAm-protected Pd NPs were stirred with the mixture of InCl_3_ (*n*_*In*_ mmol), OAm (32 mL) and OA (3.3 mL) at *T* °C for 3 h (Supplementary Table 1). Then, the organic solution was cooled to room temperature and the Pd–In alloy NPs were collected by centrifugation with ethanol and purified twice with a chloroform/ethanol (3/5 vol.%) mixed solvent.

### Synthesis of Pd–In@FeO_*y*_ NPs

The FeO_*y*_ shells were grown on the Pd–In alloy NPs by the almost same procedure as the previous report^17^, namely, injecting Fe(CO)_5_ (*n*_Fe_ mmol) into the ODE solution (63 mL) containing the Pd–In alloy NPs (0.50 mmol_Pd_), OAm (2.0 mL) and OA (3.2 mL) with stirring at 80 °C for 1 h under an N_2_ atmosphere, and the reaction temperature was increased from 80 °C to 180 °C at the heating rate of 3 °C/min (Table S1), where the amount of OAm was sensitive for the decomposition of Fe(CO)_5_ decomposition^18^. Finally, after the solution was kept at 180 °C for *t* h and cooled to room temperature, the Pd–In@FeO_y_ NPs were collected by centrifugation with ethanol, purified twice with a *n*-hexane/ethanol (3/1 vol.%) mixed solvent containing OAm (0.50 mL) and OA (0.50 mL), washed 3 times with ethanol and dried in a desiccator overnight.

### Synthesis of Pd–Pb alloy NPs

After the surface ligands on Pd seeds were exchanged as the same process as Fe–Pd–In system, the OAm-protected Pd seeds were stirred with the mixture of Pb(OAc)_2_·3H_2_O (0.3 mmol), distilled OAm (36 mL), and OA (4 mL) at 160 °C for 3 h after degassing by freeze-pump-thaw cycle (3 times). After the reaction, the organic solvent was cooled to room temperature and the Pd–Pb alloy NPs were collected by centrifugation with ethanol and purified twice with a chloroform/ethanol (3/5 vol.%) mixed solvent.

### Synthesis of Pd–Pb@FeO_*y*_ NPs

The FeO_*y*_ shells were covered on the Pd–Pb alloy NPs by injecting Fe(CO)_5_ (7.4 mmol) at 80 °C for 1 h under an N_2_ atmosphere into the mixture solution of ODE (32 mL), distilled-OAm (1.0 mL) and the Pd–Pb alloy NPs (0.25 mmol_Pd_) after degassing by freeze-pump-thaw cycle (3 times) and increasing from 80 °C to 140 °C at the heating rate of 3 °C/min. After the solution reached at 140 °C and was cooled to room temperature, the Pd–Pb@FeO_y_ NPs were collected by centrifugation with ethanol, purified twice with a *n*-hexane/ethanol (5/3 vol.%) mixed solvent containing OAm (0.50 mL) and OA (0.50 mL), washed one time with ethanol and dried in a desiccator overnight.

### Reduction-diffusion process for Pd–In@FeO_*y*_ and Pd–Pb@FeO_*y*_ NPs

The Pd–In@FeO_*y*_-NPs powders were heated at the rate of 10 °C/min, annealed at 600 °C or 800 °C for 3 h and cooled at the rate of 10 °C/min under an Ar+4% H_2_ gas flowing at 0.5 L/min. The Pd–Pb@FeO_*y*_-NPs powders were also annealed at 600 °C for 3 h via the same heating and cooling process under the same atmosphere.

### Growth of SiO_2_ shell on *A*1-PdIn_*x*_@FeO_*y*_ NPs

In a typical method^28^, the SiO_2_ shell was coated on *A*1-PdIn_*x*_@FeO_*y*_ NPs by adding TEOS (200 μL) in the cyclohexane solution (68 mL) containing *A*1-PdIn_*x*_@FeO_*y*_ NPs (0.25 mmol_Pd_), IGEPAL@CO-520 (9.6 mL) and NH_3_aq (1.6 mL) with stirring at room temperature for 16 h. After the reaction, the *A*1-PdIn_*x*_@FeO_*y*_@SiO_2_ NPs were collected by centrifugation with MeOH, purified with MeOH and twice with EtOH and dried in a desiccator overnight.

### XAFS measurement

Fe K-edge, Pd K-edge, and In K-edge EXAFS measurements were performed by using the BL01B1 beamline of SPring-8 in Japan, where the incident X-ray beam was monochromated by a Si(311) double crystal monochromator. The sample powder was mixed with BN powder and pressed into a pellet. XAFS spectra of the *L*1_2_-(Fe,In)Pd_3_ pellet were measured at 300 K, while XAFS spectra of the *Z*3-Fe(Pd,In^*d*^)_3_ pellet were measured at 10 K in a Cu folder attached on a cryostat. The EXAFS analysis was carried out by using the REX2000 Ver. 2.5 program (Rigaku Co.)^29^. In the curve-fitting analysis of the EXAFS oscillation and Fourier transform (FT) of EXAFS, the phase shift and the back-scattering amplitude function of the Fe–Fe, Fe–Pd, Pd–Pd, Pd–In and Fe–In were estimated from α-Fe (PDF#00-006-0696), *L*1_0_-FePd (PDF#03-065-9971), Pd (PDF#00-046-1043), *B*2-PdIn (PDF#03-065-4804) and the crystal information file obtained from Rietveld refinement for *Z*3-Fe(Pd,In^*d*^)_3_, respectively, by using the FEFF8 program^30^.

### TEM, HAADF-STEM and elemental-maps observations

The Pd NPs, the Pd–In alloy NPs, and the Pd–In@FeO_*y*_ NPs before and after the reduction-diffusion process were dispersed in chloroform and dropped on amorphous carbon-coated copper grids (JEOL). The TEM samples of the Fe–Pd–In ternary alloy NPs were prepared by annealing the Pd–In@FeO_*y*_ NPs on TEM grids. TEM images were recorded on a JEM1011 (JEOL) at an acceleration voltage of 100 kV. HAADF-STEM images and EDX maps were obtained by a JEM-ARM200CF at an acceleration voltage of 120 kV for the samples prepared by dropping Fe–Pd–In(Pb) ternary alloy NPs on TEM grids.

### Powder XRD measurement

The XRD patterns were recorded on a PANalytical X’Pert Pro MPD diffractometer with Cu Kα radiation (λ = 1.542 Å) at 45 kV and 40 mA. Rietveld refinements were performed by using the computer program RIETAN-2000 (ref. ^31^).

### EDX measurement and SEM observations

The EDX measurements were performed using EDAX APOLLO XF attached by scanning electron microscope (SEM, HITACH S-4800) at 20 kV, which detected Fe-K, Pd-L, In-L, and Pb-M peaks. The SEM images were observed at 30 kV.

### *M*–*H* hysteresis loop

The magnetization–magnetic field (*M*–*H*) curves of *L*1_2_-(Fe,In)Pd_3_ and *Z*3-Fe(Pd,In^*d*^)_3_ structures were obtained by a VSM (TOEI VSM-5) under a magnetic field (*H*) of –20 to 20 kOe at room temperature (~25 °C). To avoid rotating the grains during the magnetic measurements, the samples were fixed with eicosane (melting point of 36.7 °C).

### Hydrogen-storage capacity

The pressure–composition isotherms were recorded on a BELSORP-HP and measured at 30 °C and 60 °C for the sample with the Pd/Fe and Pd/In molar ratio of 65/35 at.% and 84/16 at.%, respectively (Supplementary Figs. 8 and 10).

### First-principles calculations

We performed first-principles calculations for the *Z*3- and *L*1_2_-type Fe–Pd–*M* systems within density functional theory. We used the computational code OpenMX which was based on optimized pseudopotentials and pseudo-atomic-orbital basis functions^32^. The generalized gradient approximation of Perdew-Burke-Ernzerhof was selected for an exchange-correlation functional^33^. In each calculation, we replaced one Fe or Pd atom with one *M* (*M* = Zn, Cd, Hg, Ga, In, Tl, Ge, Sn, and Pb) atom to determine the stability of the systems (Supplementary Tables 3–7). There are two different Wyckoff positions of Pd labeled by 4*i* and 1*d* that are similar to 1*b* in *Z*3-type FePd_3_. For simplicity, we noted them as *Z*3-Fe_2_(Pd_5_,*M*_1_^*i*^) and *Z*3-Fe_2_(Pd_5_,*M*_1_^*d*^). For the basis sets, we chose *s*2*p*2*d*2 configurations for Fe and *s*2*p*2*d*2*f*1 for Pd and *M*. The cut-off radius for Fe was 6.0 atomic units (a.u.) and 7.0 a.u. for Pd and In. The cut-off energy was 500 rydberg, and the *k*-point mesh was 30 × 30 × 16 and 21 × 21 × 30 for the *Z*3- and *L*1_2_-type Fe–Pd–*M* systems, respectively. Conventional cells were chosen as 1 × 1 × 1 and √2 × √2 × 1 for the *Z*3- and *L*1_2_-type Fe–Pd–*M* systems, respectively. These conventional cells had the same number and kinds of atoms in a unit cell, which allowed us to compare the energy difference between these systems easily. Obtained calculation results were at 0 K and we did not consider the reaction paths of how the In-doped FePd_3_ structure was fabricated because of the limitation of the first-principles calculations. The formation energies of *L*1_2_-(Fe_2-*x*_,In_*x*_)Pd_6_ and *Z*3-Fe_2_(Pd_6-*x*_,In_*x*_^*d*^) (*E*_*L*12_ and *E*_*Z*3_, respectively) were estimated by comparing *E*_*L*12_ and *E*_*Z*3_ under the same number and kinds of atoms as follows (0 < *x* < 1);

2 $${E}_{L12}=x\times E[L{1}_{2}-({{{{{{\rm{Fe}}}}}}}_{1},{{{{{{\rm{In}}}}}}}_{1}){{{{{{\rm{Pd}}}}}}}_{6}]+(1-x)\times E[L{1}_{2}-{{{{{{\rm{Fe}}}}}}}_{2}{{{{{{\rm{Pd}}}}}}}_{6}]+x\times \mu [{{{{{\rm{Fe}}}}}}]$$

and

3 $${E}_{Z3}=x\times E[Z3-{{{{{{\rm{Fe}}}}}}}_{2}({{{{{{\rm{Pd}}}}}}}_{5},{{{{{{{\rm{In}}}}}}}_{1}}^{d})]+(1-x)\times E[Z3-{{{{{{\rm{Fe}}}}}}}_{2}{{{{{{\rm{Pd}}}}}}}_{6}]+x\times \mu [{{{{{\rm{Pd}}}}}}]$$

where *E*[phase] is the *E*_form_ of each phase. Furthermore, in order to analyse the magnetic anisotropy of *Z*3- and *L*1_2_-type Fe–Pd–In in Fig. 4, we compared the total energies obtained by non-collinear calculations in which spin directions on each atom were fixed within 0< *θ*(°) <90 and 0< *φ*(°) <45 (i.e., from [001] to [100] at *φ* = 0 and from [100] to [110] at *θ* = 0). Conventional cells in these non-collinear calculations were expanded to 2 × 2 × 1 and 2 × 2 × 2 for *Z*3-Fe_8_(Pd_20_,In_4_^*d*^) and *L*1_2_-(Fe_7_,In_1_)Pd_24_ systems, respectively (Supplementary Table 8).

## Supplementary information

Supplementary Information

## Data Availability

All data supporting the findings of this study are available from the corresponding author on request.
