# Supplementary material for: Inter-element miscibility driven stabilization of ordered pseudo-binary alloy
Source: Nat Commun. 2022 Feb 24;13:1047. doi: 10.1038/s41467-022-28710-0 (PMC8873263; doi:10.1038/s41467-022-28710-0)
Supplement: Supplementary file 1 — Supplementary Information [file 41467_2022_28710_MOESM1_ESM.pdf]

Supplementary Information for

Inter-element miscibility for unprecedented ordered alloys

Kenshi Matsumoto, Ryota Sato, Yasutomi Tatetsu, Ryo Takahata, Seiji Yamazoe, Miho Yamauchi, Yuji Inagaki, Yoichi Horibe, Masaki Kudo, Takaaki Toriyama, Mitsunari Auchi, Mitsutaka Haruta, Hiroki Kurata, Toshiharu Teranishi*.

Correspondence to: teranisi@scl.kyoto-u.ac.jp

This PDF file includes:

Supplementary Figs. 1–10
Supplementary Tables 1–8

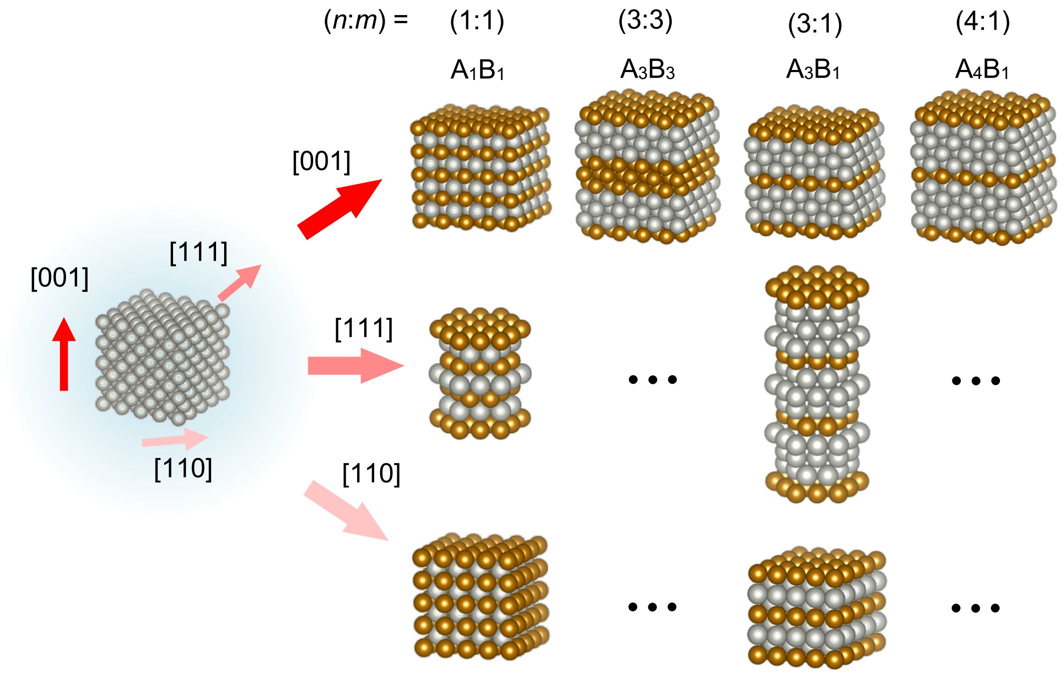

Supplementary Fig. 1 | Candidates of mille-feuille binary alloy (A_nB_m) structures. A number of layered structures based on the face-centred-cubic framework are available by alternating *n* layered A and *m* layered B along different crystal directions such as [001], [111] and [110].

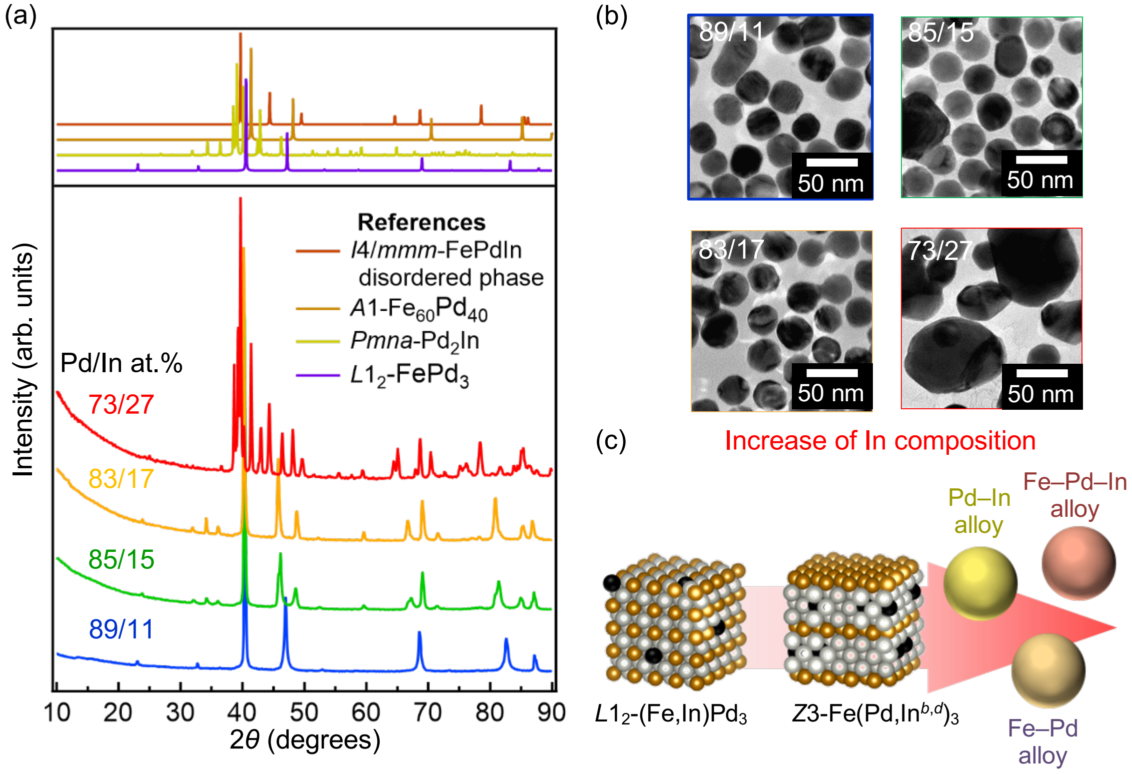

Supplementary Fig. 2 | Change in crystal structure depending on the In composition. a,b, Powder XRD patterns (**a**) and TEM images (**b**) of the samples with the Pd/Fe atomic ratio of approximately 70/30 after the reductive annealing at 800 °C for 3 h, where the lattice parameters (*a* and *c*) of the *I*4/*mmm*-Fe–Pd–In disordered phase are 2.88 and 3.68 Å. **c,** Schematic of the crystal structure change by different In composition.

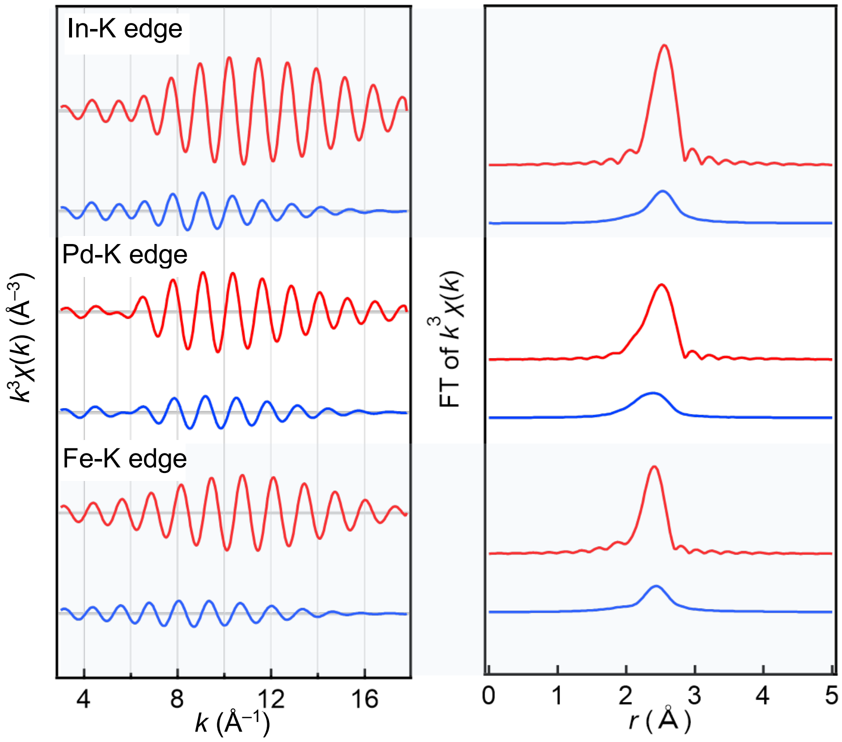

Supplementary Fig. 3 | EXAFS analysis of $L1_2$-type and $Z3$-type structures. EXAFS oscillation ($k^3\chi(k)$) of the $L1_2$-type and $Z3$-type phases in Fe-, Pd- and In-K edges and the Fourier transformation (FT) of $k^3\chi(k)$, where these analyses were carried out at $3.00< k <17.8$ and $1.93< r <2.98$ at the Fe-K edge, $3.00< k <17.8$ and $1.38< r <3.15$ at the Pd-K edge and $3.00< k <17.8$ and $1.38< r <3.13$ at the In-K edge. Red and blue curves are fitting curves for the $Z3$-type and $L1_2$-type phases, respectively.

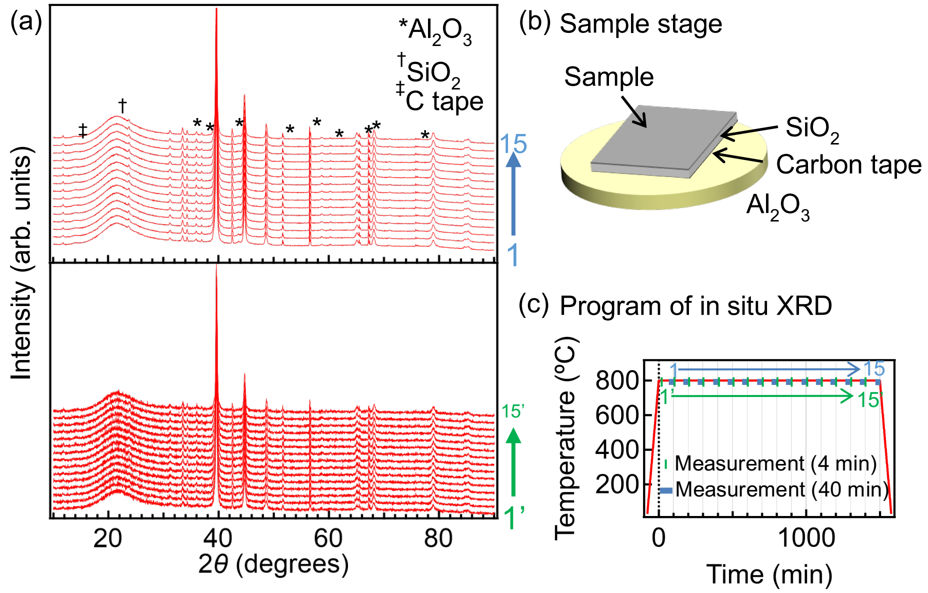

Supplementary Fig. 4 | Investigation on the stability of *Z*3-type phase. a, In situ XRD measurements for *A*1-$PdIn_x$@FeO_y@SiO_2 core@shell@shell nanoparticles with the Pd/In/Fe composition of 62/13/25 at.% during the reductive annealing at 800 °C. **b,** Sample stage for in situ XRD measurements. **c,** Temperature control program for the in situ XRD measurements.

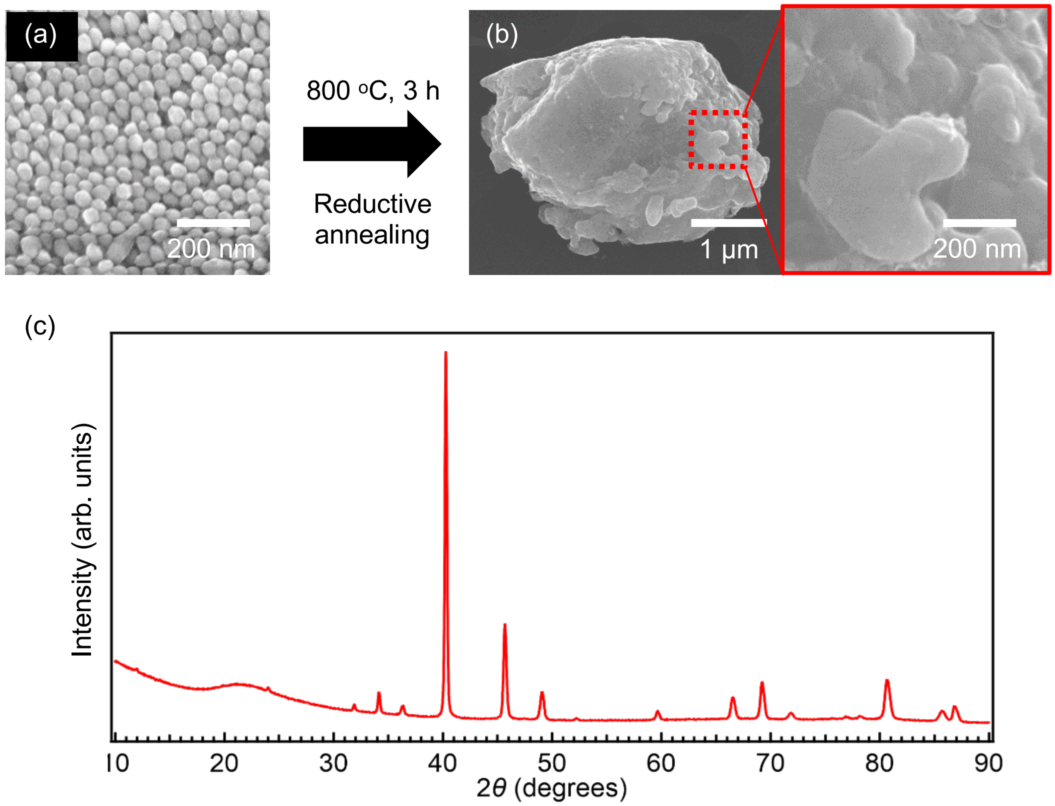

Supplementary Fig. 5 | Synthesis of *Z*3-Fe(Pd,In^{d})$_3$ microparticles. a, SEM images of Pd–In@FeO$_x$ NPs with Pd/In/Fe composition of 61/11/28 at.% after annealing under air at 300 °C for 3 h. **b,** SEM images of microparticle formed by the reductive annealing for **a**. **c,** Powder XRD patterns of **b**.

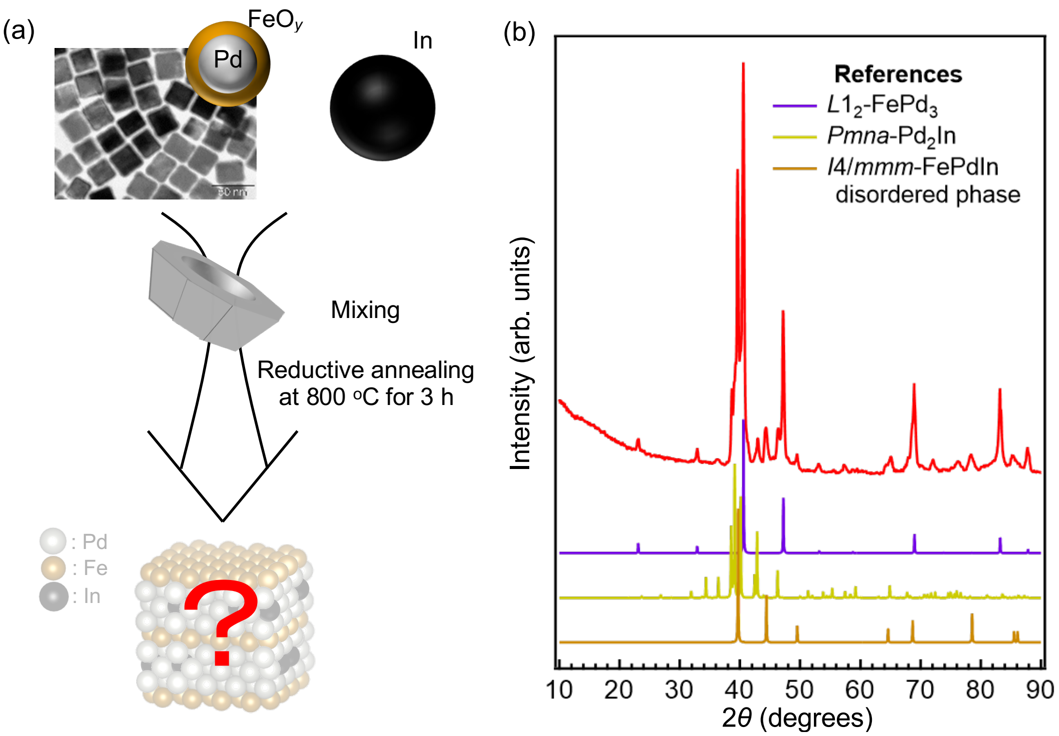

Supplementary Fig. 6 | Reductive annealing of the mixture of Pd@FeO$_y$ NPs and In powder. **a,** Schematic of the synthesis process. The Pd/In and Pd/Fe atomic ratios of the mixture were 82/18 and 74/26, respectively. As the melting point of In metal is 156.6 °C, the Fe–Pd alloy NPs can be covered with an In-liquid matrix during the reductive annealing at 800 °C for 3 h. **b,** Powder XRD patterns of the mixture after the reductive annealing. The lattice parameters (*a* and *c*) of *I*4/*mmm*-Fe–Pd–In disordered phase are 2.88 and 3.68 Å, respectively.

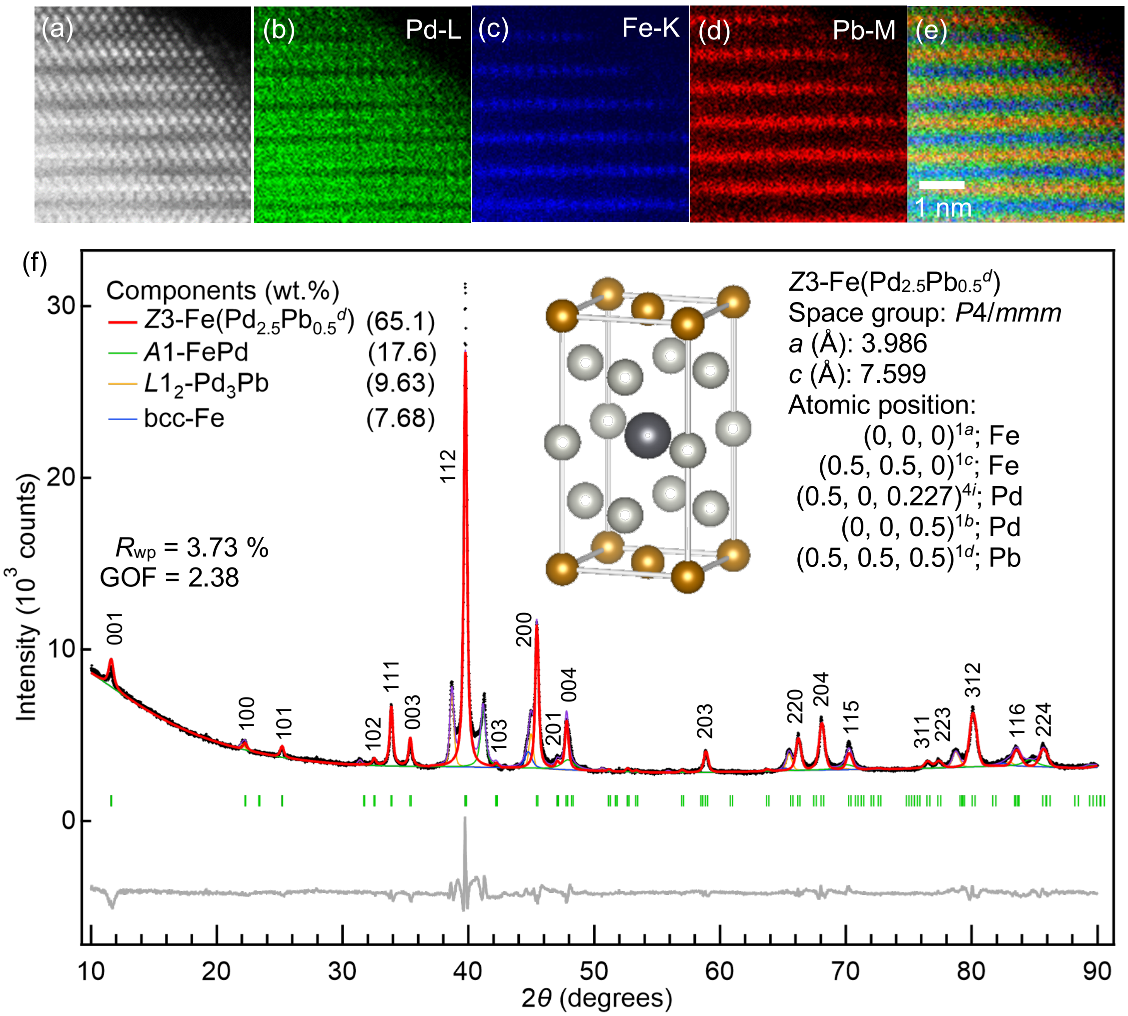

Supplementary Fig. 7 | Characterization of Fe–Pd–Pb ternary alloy NPs. a–d, HAADF-STEM image (**a**) and the elemental maps (Pd-L (**b**), Fe-K (**b**) and Pb-M (**c**)) of *Z*3-type structure. **e,** Overlay of **b–d**. **f**, Rietveld refinement for powder XRD patterns of Fe–Pd–Pb ternary alloy NPs with the Pd/Pb/Fe composition of 50/9/41 at.%, where the black markers are raw data, the purple lines are the sum of the fitting curves, the grey line is a difference curve of raw data and the fitting curve and the green bars stand for the whole diffraction-peak positions. R_{wp} and GOF are a reliability factor and goodness of fit, respectively. Pb coordination of *Z*3-type structure could be detected not only by EDX elemental maps at atomic resolution but also by powder XRD patterns, because Pb possessed large electron density, compared with Fe and Pd.

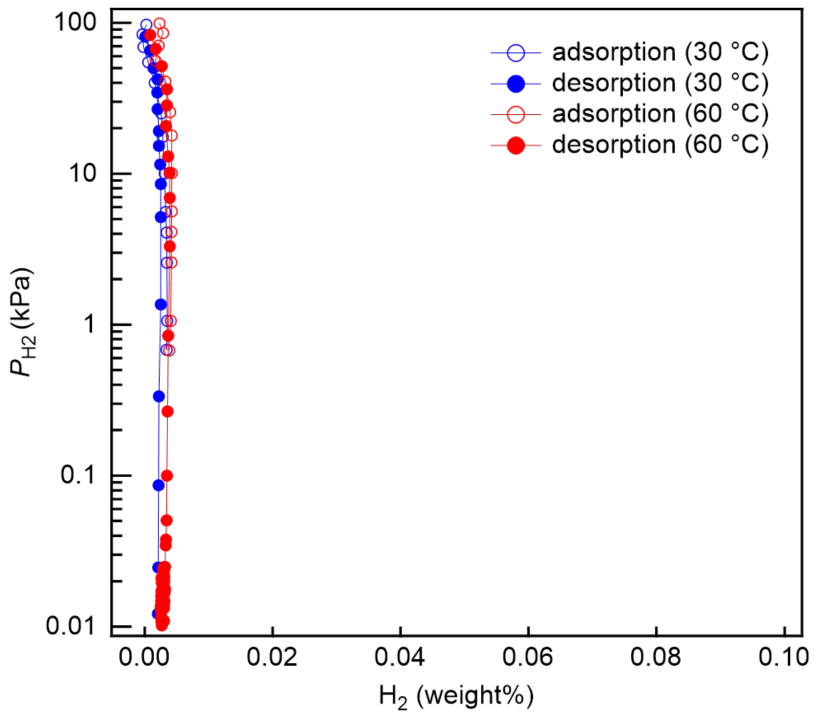

Supplementary Fig. 8 | Hydrogen pressure-composition isotherms for *Z*3-type structure. We measured the hydrogen pressure-composition isotherms at 30 °C and 60 °C following hydrogen adsorption and desorption at each temperature.

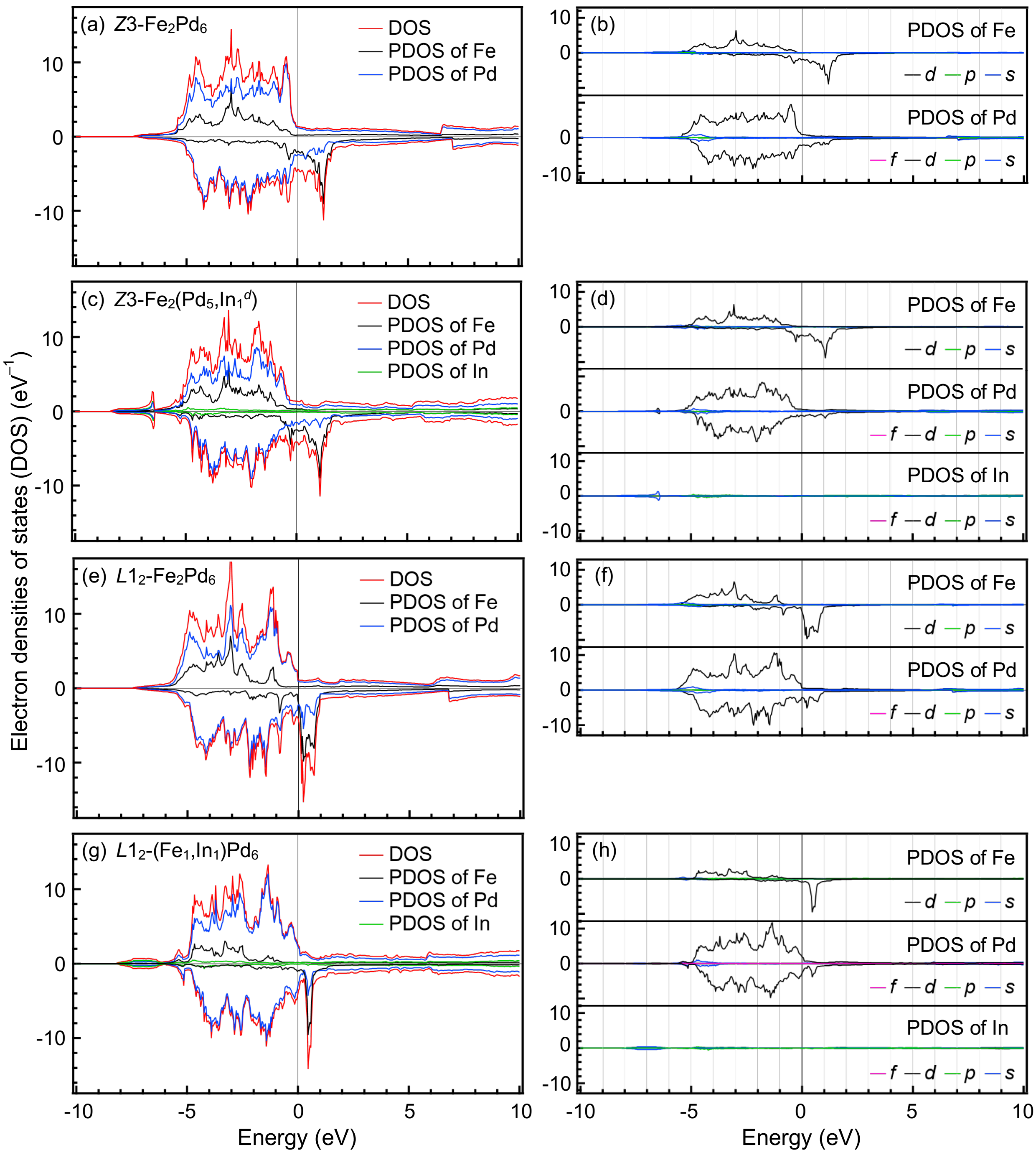

Supplementary Fig. 9 | Partial DOS (PDOS) of $L1_2$- and $Z3$-Fe_2Pd_6, $Z3$-$Fe_2(Pd_5,In_1{}^{d})$ and $L1_2$-$(Fe_1,In_1)Pd_6$ structures. a,c,e,g, DOS and PDOS of each element in the $Z3$-Fe_2Pd_6 (**a**), $Z3$-$Fe_2(Pd_5,In_1{}^{d})$ (**c**), $L1_2$-Fe_2Pd_6 (**e**) and $L1_2$-$(Fe_1,In_1)Pd_6$ (**g**) structures. **b,d,f,h,** PDOS of each orbital in the $Z3$-Fe_2Pd_6 (**b**), $Z3$-$Fe_2(Pd_5,In_1{}^{d})$ (**d**), $L1_2$-Fe_2Pd_6 (**f**) and $L1_2$-$(Fe_1,In_1)Pd_6$ (**h**) structures. The Fermi energy is set at 0 eV.

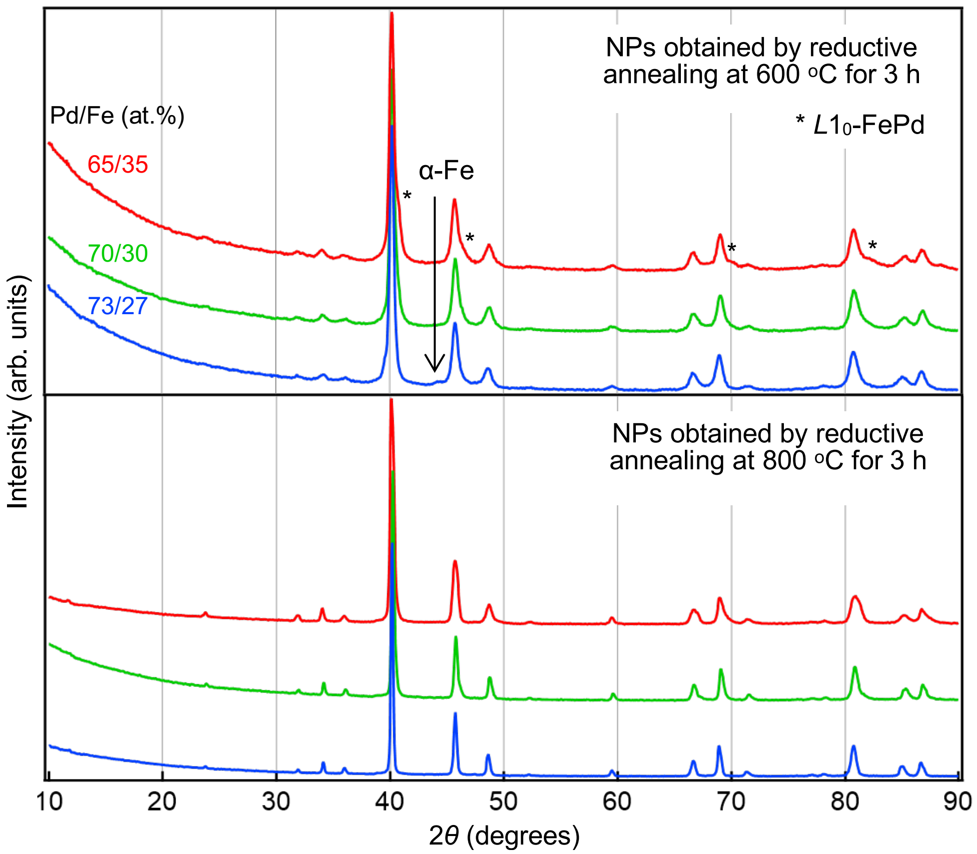

Supplementary Fig. 10 | Thermodynamically stable phases of Fe–Pd–In ternary alloy NPs depending on the Fe composition and the annealing temperatures. The samples for powder XRD measurements were obtained by the reductive annealing of the $PdIn_x@FeO_y$ NPs with Pd/In ≈ 83/17 at.% at 600 °C or 800 °C for 3 h. The NPs (Pd/Fe = 65/35 and 73/27 at.%) obtained by the reductive annealing at 600 °C for 3 h contained not only the *Z*3-type phase but also the $L1_0$-FePd (a = 3.88 and c = 3.67 Å) and α-Fe phases, respectively, which indicates that the existence of minor phases causes a difference in the compositions of the final products and the main phase such as the $L1_2$-(Fe,In)Pd_3 and *Z*3-Fe(Pd,In^d)$_3$ phases.

Supplementary Table 1 | Synthetic conditions and crystals structures of the Fe–Pd–In ternary alloy NPs obtained by the reductive annealing at 800 °C for 3 h.

Pd–In NPs		Pd–In@FeO$_y$ NPs		Final products	
T (°C)	n_{In} (mmol)	t (h)	n_{Fe} (mmol)	Final phases	Comsption of Pd:In:Fe (at.%)
330	0.25	0.30	8.9	*I*4/*mmm* + *Pmna* + *A*1	56:20:24
315	0.10	1.0	13	*Z3*	63:14:23
315	0.10	2.0	30	*Z*3	58:11:31
315	0.10	2.0	22	*Z*3	62:12:26
315	0.10	1.2	15	*Z*3	61:11:28
300	0.05	1.0	16	*Z*3	63:11:26
300	0.05	1.5	15	$L1_2$	64:8:28

Supplementary Table 2 | EXAFS-oscillation analysis of $L1_2$-type and $Z3$-type structures in Fe-, Pd- and In-K edges. a, Fitting parameters of EXAFS oscillation, where N is the coordination number, r is the atomic distance, σ^2 is the Debye–Waller factor and R is the reliability factor calculated from the formula, $(\Sigma(k^3\chi^{\mathrm{data}}(k)-k^3\chi^{\mathrm{fit.}}(k))^2)^{1/2}/(\Sigma(k^3\chi^{\mathrm{data}}(k))^2)^{1/2}$ (ref. 29). **b,** Candidates of In sites in the $L1_2$-type and $Z3$-type structures, where $r_{\mathrm{In–Pd}}$ and $r_{\mathrm{In–Fe}}$ refer to the In–Pd and In–Fe distances, respectively and $N_{\mathrm{In@Pd}}$ and $N_{\mathrm{In@Fe}}$ are the coordination numbers of Pd and Fe around an In atom, respectively.

(a)

	Fe-K	***N***	***r* (Å)**	**σ^2 × 10^3 (Å^2)**	***R*%**
$L1_2$	Fe-Pd	5.57	2.64	9.22	14.9
$Z3$	Fe-Pd	6.59	2.62	4.49	7.16
	Fe-Fe	3.43	2.77	7.23	
	**Pd-K**	***N***	***r* (Å)**	**σ^2 × 10^3 (Å^2)**	***R*%**
$L1_2$	Pd-Pd(In)	4.98	2.76	7.74	7.26
	Pd-Fe	1.69	2.61	4.49	
$Z3$	Pd-Pd(In)	6.38	2.82	3.60	8.34
	Pd-Fe	2.85	2.64	2.81	
	**In-K**	***N***	***r* (Å)**	**σ^2 × 10^3 (Å^2)**	***R*%**
$L1_2$	In-Pd(In)	11.9	2.77	9.60	9.71
$Z3$	In-Pd(In)	12.9	2.82	3.84	9.65

(b)

**Candidates of In sites**	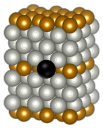	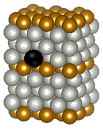	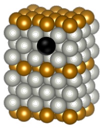	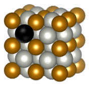	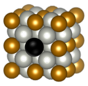
$r_{\mathrm{In–Pd}}$ (Å) ($N_{\mathrm{In@Pd}}$)	2.63 (8)	~2.81 (8)	~2.82 (12)	2.73 (8)	2.73 (12)
$r_{\mathrm{In–Fe}}$ (Å) ($N_{\mathrm{In@Fe}}$)	2.80 (4)	2.63 (4)	0	2.73 (4)	0

Supplementary Table 3 | Crystal structure and the formation energy of $L1_2$-$Fe_2(Pd_5,X_1)$ and $L1_2$-Fe_2Pd_6 phases obtained by first-principles calculations.

**$L1_2$-$Fe_2(Pd_5,X_1)$** 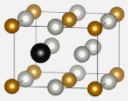		***a*** **(Å)**	***b*** **(Å)**	***c*** **(Å)**	***α*** **(°)**	***β*** **(°)**	***γ*** **(°)**	**E_{L12} (eV atom^{-1})**
$L1_2$-Fe_2Pd_6		5.5096	5.5096	3.8959	90.000	90.000	90.000	−0.09150
	Zn	5.4245	5.4245	4.0079	90.000	90.000	97.028	−0.17944
	Ga	5.4557	5.4558	4.0032	90.000	90.000	97.643	−0.22429
	Ge	5.4699	5.4699	4.0029	90.000	90.000	97.753	−0.16321
	Cd	5.5306	5.5306	4.0336	90.000	90.000	94.617	−0.10614
X	In	5.5581	5.5581	4.0351	90.000	90.000	94.994	−0.15442
	Sn	5.5747	5.5747	4.0199	90.000	90.000	94.298	−0.15720
	Hg	5.5699	5.5698	4.0092	90.000	90.000	93.468	−0.04174
	Tl	5.6179	5.6186	4.0386	90.000	90.000	94.689	−0.01231
	Pb	5.6579	5.6579	4.0111	90.000	90.000	93.392	−0.02361

Supplementary Table 4 | Crystal structure and the formation energy of $L1_2$-(Fe_1,X_1)Pd_6 phase obtained by first-principles calculations.

$L1_2$-(Fe_1,X_1)Pd_6 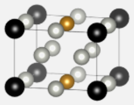		a (Å)	b (Å)	c (Å)	α (°)	β (°)	γ (°)	E_{L12} (eV atom^{-1})
X	Zn	5.5400	5.5400	3.8857	90.000	90.000	90.000	−0.20462
	Ga	5.5378	5.5378	3.9133	90.000	90.000	90.000	−0.26568
	Ge	5.5283	5.5283	3.9464	90.000	90.000	90.000	−0.21415
	Cd	5.6761	5.6761	3.8685	90.000	90.000	90.000	−0.16792
	In	5.6600	5.6600	3.9193	90.000	90.000	90.011	−0.24524
	Sn	5.6381	5.6381	3.9633	90.000	90.000	90.000	−0.26108
	Hg	5.7254	5.7254	3.8476	90.000	90.000	90.000	−0.09702
	Tl	5.7581	5.7581	3.8711	90.000	90.000	90.000	−0.10422
	Pb	5.7363	5.7363	3.9359	90.000	90.000	90.000	−0.13997

Supplementary Table 5 | Crystal structure and the formation energy of *Z*3-$Fe_2(Pd_5,X_1{}^{d})$ and *Z*3-Fe_2Pd_6 phases obtained by first-principles calculations.

**$Z3$-$Fe_2(Pd_5,X_1{}^{d})$** 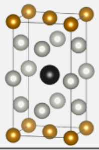		***a*** **(Å)**	***b*** **(Å)**	***c*** **(Å)**	***α*** **(°)**	***β*** **(°)**	***γ*** **(°)**	**E_{Z3} (eV atom^{-1})**
$Z3$-Fe_2Pd_6		3.9021	3.9021	7.7320	90.000	90.000	90.000	−0.08917
X	Zn	3.9096	3.9096	7.6192	90.000	90.000	90.000	−0.20351
	Ga	3.9235	3.9235	7.6026	90.000	90.000	90.000	−0.26157
	Ge	3.9125	3.9125	7.6841	90.000	90.000	90.000	−0.20484
	Cd	3.9664	3.9664	7.7378	90.000	90.000	90.000	−0.17803
	In	3.9898	3.9898	7.7000	90.000	90.000	90.000	−0.24785
	Sn	3.9848	3.9848	7.7411	90.000	90.000	90.000	−0.25524
	Hg	3.9715	3.9715	7.8020	90.000	90.000	90.000	−0.11460
	Tl	4.0152	4.0152	7.7786	89.999	89.999	90.000	−0.11527
	Pb	4.0135	4.0135	7.8561	90.000	90.000	90.000	−0.14096

Supplementary Table 6 | Crystal structure and the formation energy of $Z3$-$Fe_2(Pd_5,X_1^{i})$ phase obtained by first-principles calculations.

$Z3$-$Fe_2(Pd_5,X_1^{i})$ 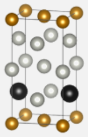		a (Å)	b (Å)	c (Å)	α (°)	β (°)	γ (°)	E_{Z3} (eV atom^{-1})
X	Zn	3.8652	3.8652	7.7872	90.000	90.000	90.000	−0.15131
	Ga	3.8821	3.8821	7.7672	90.000	90.000	90.000	−0.20167
	Ge	3.9000	3.9000	7.7307	90.000	90.000	90.000	−0.14945
	Cd	3.9214	3.9214	7.9298	90.000	90.000	90.000	−0.09685
	In	3.9367	3.9367	7.9352	90.000	90.000	90.000	−0.15453
	Sn	3.9509	3.9509	7.8951	90.000	90.000	90.000	−0.16658
	Hg	3.9471	3.9471	7.8936	90.000	90.000	90.000	−0.04107
	Tl	3.9701	3.9701	7.9614	89.999	89.999	90.000	−0.01852
	Pb	3.9832	3.9832	8.0035	90.000	90.000	90.000	−0.03761

Supplementary Table 7 | Crystal structure and the formation energy of *Z*3-(Fe_1,X_1)Pd_6 phase obtained by first-principles calculations.

*Z*3-(Fe_1,X_1)Pd_6 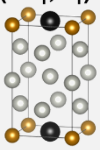		*a* (Å)	*b* (Å)	*c* (Å)	*α* (°)	*β* (°)	*γ* (°)	E_{Z3} (eV atom^{-1})
X	Zn	4.0068	4.0068	7.4298	90.000	90.000	90.000	−0.20039
	Ga	4.0525	4.0525	7.3088	89.998	89.995	89.986	−0.25244
	Ge	4.0263	4.0263	7.4114	90.000	90.000	90.001	−0.18662
	Cd	4.0531	4.0531	7.6110	90.000	90.000	90.000	−0.10848
	In	4.1107	4.1107	7.4658	90.000	89.997	90.000	−0.17092
	Sn	4.0494	4.0494	7.6765	90.000	90.000	90.000	−0.19008
	Hg	4.0448	4.0448	7.7133	90.000	90.000	90.000	−0.04092
	Tl	4.1714	4.1714	7.4156	90.000	90.000	89.997	−0.01932
	Pb	4.0809	4.0809	7.7501	90.000	90.000	90.000	−0.03929

Supplementary Table 8 | Crystal structure and magnetic anisotropy energy (MAE) of $L1_2$-$(Fe_7,In_1)Pd_{24}$ and $Z3$-$Fe_8\,(Pd_{20},In_4^d)$ phases obtained by non-collinear calculations.

	a (Å)	*b* (Å)	*c* (Å)	*α* (°)	*β* (°)	*γ* (°)	MAE (meV atom^{-1})
$L1_2$-$(Fe_7,In_1)Pd_{24}$ 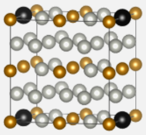	7.8226	7.6229	7.8229	90.003	90.001	90.001	−0.00138
$Z3$-$Fe_8(Pd_{20},In_4^d)$ 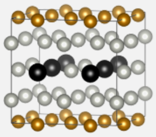	8.0080	8.0080	7.6950	89.998	89.999	90.000	−0.21310
